# Supplementary figures and images for: Neural Development Features: Spatio-Temporal Development of the Caenorhabditis elegans Neuronal Network
Source: PLoS Comput Biol. 2011 Jan 6;7(1):e1001044. doi: 10.1371/journal.pcbi.1001044 (PMC3017107; doi:10.1371/journal.pcbi.1001044)

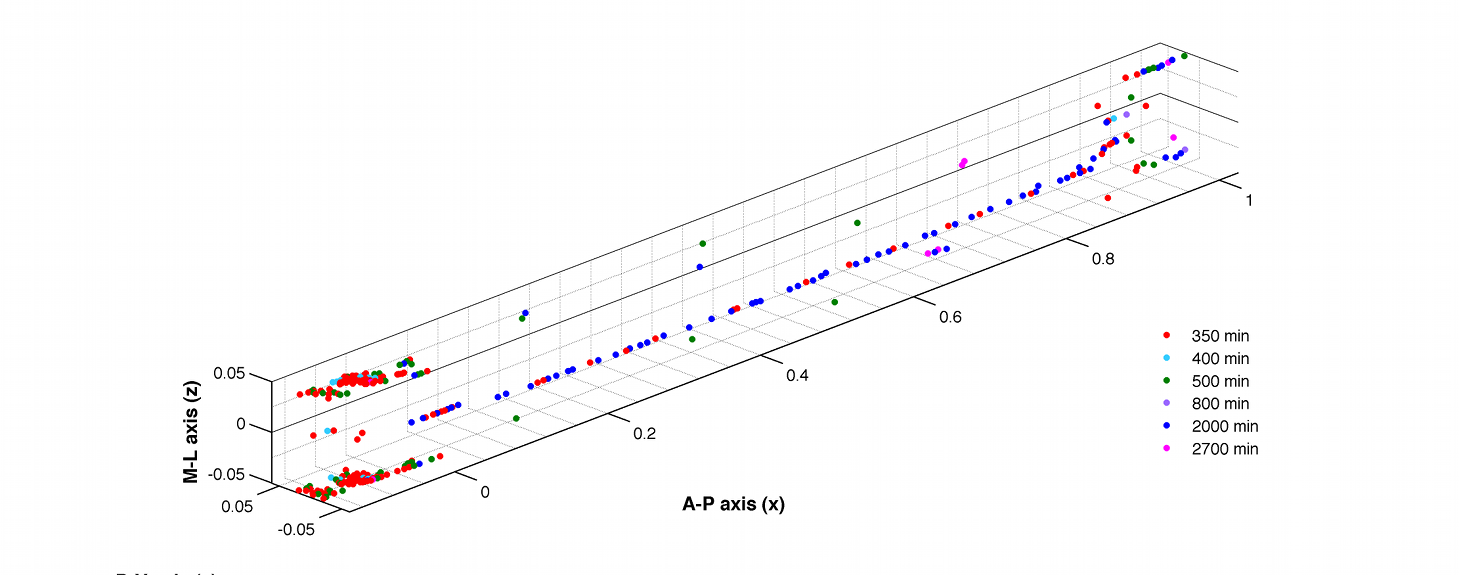

Supplement: Figure S1 — Neurons formed at successive stages of growth. Neuron positions are shown at the adult stage (orientations are along the Anterior (A)-Posterior (P), and Dorsal (D)-Ventral (V) axes). Note that some ventral cord neurons are established early whereas most occur at late stages of development. (0.31 MB TIF) [file pcbi.1001044.s001.tif]

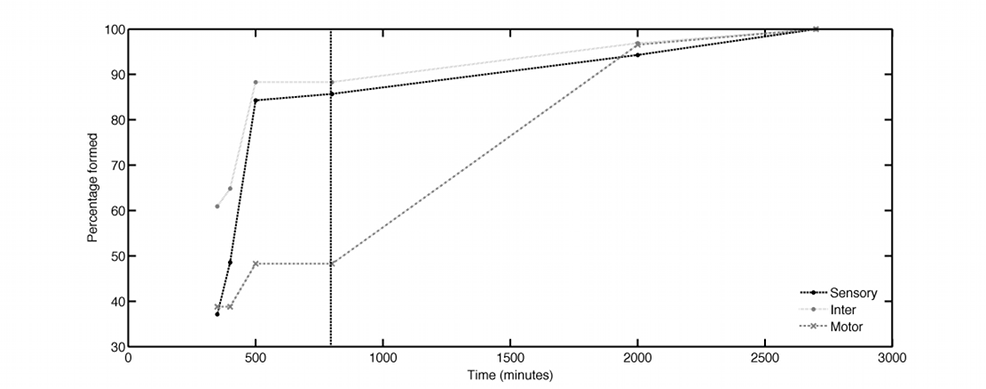

Supplement: Figure S2 — Growth pattern of sensory, motor and inter neurons. The vertical dashed line indicates the approximate time of hatching. The plot shows the percentage of neurons formed at each time step for the three neurons types (sensory, inter, and motor neurons). Note that while sensory and inter-neurons are formed early on, less than fifty percent of the motor neurons are formed at the time of hatching (800 minutes). By excluding poly-modal neurons, the percentage drops further to around 30%. (0.13 MB TIF) [file pcbi.1001044.s002.tif]

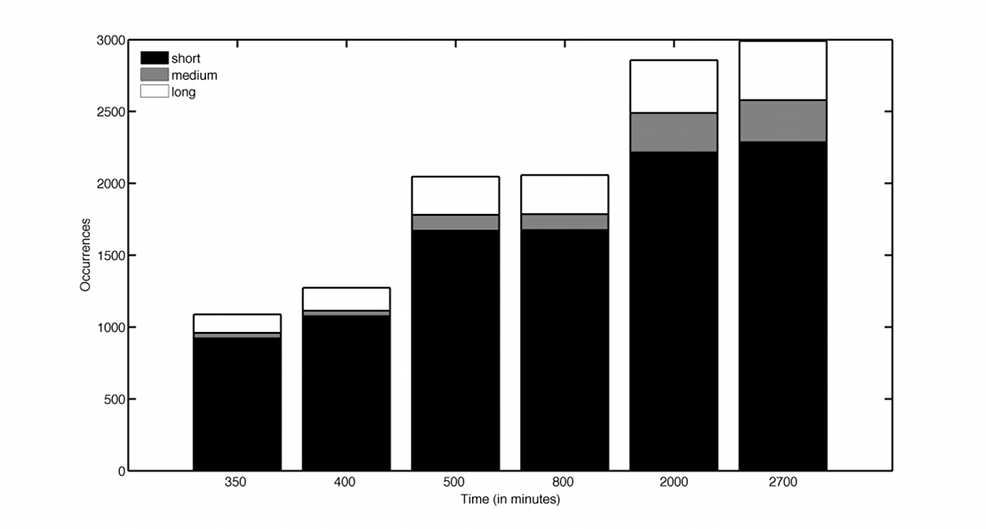

Supplement: Figure S3 — Distribution of approximated metric connection lengths during development as a reflection of neuron births. The metric lengths correspond to the adult and the presence at a particular development stage signifies the existence of both neurons that are connected in the adult. The proportion of small-, medium-, and long-distance connection pairs are shown in each bar and identified in the legend (See also Figure S1). (0.13 MB TIF) [file pcbi.1001044.s003.tif]

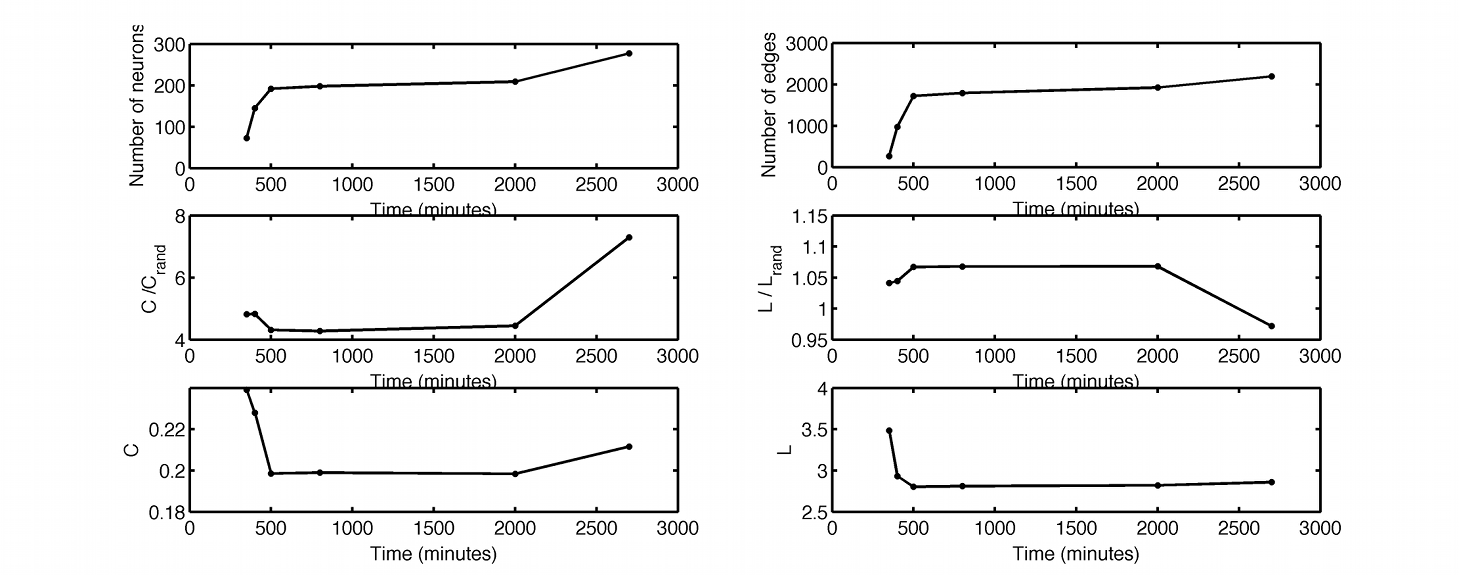

Supplement: Figure S4 — Change in topological network organization during development represented at each discrete time stage. (A) The total number of neurons formed by the end of each of the discrete time steps (black dots) superimposed on the actual birth times of neurons (gray dots) (C) Ratio of the clustering coefficient of the actual network compared to a random network (C/Crand). (D) Ratio of the characteristic path length of the actual network compared to a random network (L/Lrand). (E) and (F) absolute values of the clustering coefficient C and characteristic path length L, respectively. Time of hatching (approximately 840 minutes) is soon after the third discrete time step (800 minutes). (0.29 MB TIF) [file pcbi.1001044.s004.tif]
