# Supplementary material for: Neural Development Features: Spatio-Temporal Development of the Caenorhabditis elegans Neuronal Network
Source: PLoS Comput Biol. 2011 Jan 6;7(1):e1001044. doi: 10.1371/journal.pcbi.1001044 (PMC3017107; doi:10.1371/journal.pcbi.1001044)
Supplement: Table S1 — Number of short, medium and long-length connections for each type of synapse (electrical synapse, chemical synapse, or a combination of both features). (0.03 MB PDF) [file pcbi.1001044.s005.pdf]

Table S1. Number of short, medium and long-length connections for each type of synapse (electrical synapse, chemical synapse, or a combination of both features).

	Short	Medium	Long	Total
Gap-junction	546	66	40	652
Chemical synapse	1495	180	287	1962
Combination	260	52	64	376
Total	2301	298	391	2990
